# The bile acid receptor GPBAR1 (TGR5) is expressed in human gastric cancers and promotes epithelial-mesenchymal transition in gastric cancer cell lines

**DOI:** 10.18632/oncotarget.10477

**Published:** 2016-07-07

**Authors:** Adriana Carino, Luigina Graziosi, Claudio D'Amore, Sabrina Cipriani, Silvia Marchianò, Elisabetta Marino, Angela Zampella, Mario Rende, Paolo Mosci, Eleonora Distrutti, Annibale Donini, Stefano Fiorucci

**Affiliations:** ^1^ Dipartimento di Scienze Chirurgiche e Biomediche, Università degli Studi di Perugia, Perugia, Italy; ^2^ Azienda Ospedaliera di Perugia, Perugia, Italy; ^3^ Dipartimento di Medicina, Università degli Studi di Perugia, Perugia, Italy; ^4^ Dipartimento di Farmacia, Università di Napoli, Napoli, Italy; ^5^ Dipartimento di Medicina Veterinaria, Università degli Studi di Perugia, Perugia, Italy

**Keywords:** gastric cancer, TGR5, bile acids, epithelial-mesenchymal transition

## Abstract

GPBAR1 (also known as TGR5) is a bile acid activated receptor expressed in several adenocarcinomas and its activation by secondary bile acids increases intestinal cell proliferation. Here, we have examined the expression of GPBAR1 in human gastric adenocarcinomas and investigated whether its activation promotes the acquisition of a pro-metastatic phenotype. By immunohistochemistry and RT-PCR analysis we found that expression of GPBAR1 associates with advanced gastric cancers (Stage III-IV). GPBAR1 expression in tumors correlates with the expression of N-cadherin, a markers of epithelial-mesenchymal transition (EMT) (r=0.52; P<0.01). Expression of GPBAR1, mRNA and protein, was detected in cancer cell lines, with MKN 45 having the higher expression. Exposure of MKN45 cells to GPBAR1 ligands, TLCA, oleanolic acid or 6-ECDCA (a dual FXR and GPBAR1 ligand) increased the expression of genes associated with EMT including *KDKN2A, HRAS, IGB3, MMP10 and MMP13* and downregulated the expression of *CD44 and FAT1* (P<0.01 versus control cells). GPBAR1 activation in MKN45 cells associated with EGF-R and ERK1 phosphorylation. These effects were inhibited by DFN406, a GPBAR1 antagonist, and cetuximab. GPBAR1 ligands increase MKN45 migration, adhesion to peritoneum and wound healing. Pretreating MKN45 cells with TLCA increased propensity toward peritoneal dissemination *in vivo*. These effects were abrogated by cetuximab. In summary, we report that GPBAR1 is expressed in advanced gastric cancers and its expression correlates with markers of EMT. GPBAR1 activation in MKN45 cells promotes EMT. These data suggest that GPBAR1 antagonist might have utility in the treatment of gastric cancers.

## INTRODUCTION

Gastric adenocarcinomas, one the most challenging cancers of the gastrointestinal tract to treat, representing the second leading cause of cancer mortality worldwide [1]. The only treatment that offers a potential cure is complete resection of the tumor. However, the majority of patients are diagnosed at advanced stages and have a poor prognosis. Thought first-line chemotherapy for advanced gastric cancer prolongs overall survival, the median survival of advanced gastric cancer patients who received palliative chemotherapy is approximately 7 to 11 months with a global 5-year survival rate of 20% [2, 3]. Tumor characteristics such as invasion and metastasis are the primary causes of treatment failure in advances stages.

Bile acids are steroid molecules generated in the liver by cholesterol metabolism [4]. In addition to their role in nutrients absorption bile acids function as signaling hormones by activating a family of receptors that includes nuclear receptors such as the Farnesoid-x-receptor (FXR) [6], Constitutive Androstane Receptor (CAR), [7], Pregnane-x-receptor (PXR) [8], liver-x-receptor (LXR) [9] and the Vitamin D receptor (VDR), and G-protein coupled receptors (i.e. TGR5 or GPBAR1) [10, 11]. Bile acids activate these receptors with a different rank of potency [12, 13], with primary bile acids functioning as preferential ligands for FXR and secondary bile acids for GPBAR1. GPBAR1 is a member of the rhodopsin-like superfamily of G-protein-coupled receptor. GPBAR1 is highly represented in the gastrointestinal tract and non-parenchymal liver cells oversighting on a variety of homeostatic and regulatory functions [9–12]. Once activated by secondary bile acids (i.e. litocholic acid (LCA) and deoxycholic acid (DCA) and their tauro- and glyco-conjugated forms) GPBAR1 modulates multiple targets by genomic and non-genomic effects [9, 10]. GPBAR1 is expressed in epithelial, endocrine [11] and neuronal cells, in the stomach and intestine, and mice harboring a disrupted GPBAR1 are more prone to develop a severe intestinal inflammation [14].

Experimental data have implicated bile acids in gastrointestinal carcinogenesis [15–17]. Bile acid-induced trans-activation of Epidermal Growth factor (EGF) receptor (EGF-R) leads to the overexpression of cyclooxygenase (COX)2 in colon cancer cells [18], increasing cells proliferation and angiogenesis. In human gastric carcinoma cell line AGS, DCA activates ERK1/2, MAPK and causes a GPBAR1-dependent trans-phosphorylation of the EGF-R [19]. In this cell line, GPBAR1gene silencing by specific small interfering RNA (siRNA), suppresses DCA-induced trans-activation of EGF-R and ERK1/2 phophorylation and potentiates apoptosis [19]. Additionally, an increased expression of TGR5 (GPABR1) is associated to a reduced life expectancy in patient with gastric and esophageal adenocarcinomas [20, 21] suggesting that inhibition of this receptor might be a potential target for prevention and/or treatment of these and other tumors [22]. In contrast, other studies demonstrate that activation of GPBAR1 reduces gastric cancer proliferation suggesting a protective role of this receptor against gastric cancer dissemination [23].

Metastasis is a multi-step process that involves an epithelial–mesenchymal transition (EMT) in which polarized epithelial cells are converted to mesenchymal cells [24, 25], a phenotype with greater invasive and migratory capacity [24, 25]. The EMT is a reversible process that often occurs at the invasive front of many metastatic cancers [26] and involves profound phenotypic changes such as the loss of cell-cell adhesion, and cell polarity and the acquisition of migratory and invasive properties. A growing body of evidence support the notion that aberrant activation of EMT is a critical event in the formation of metastasis in various tumors [25], including gastric cancer [24, 25]. In this study we have investigated whether GPBAR1 is involved in gastric cancer dissemination.

## RESULTS

### Expression of bile acid activated receptors in human gastric cancers

**Figure 1:**
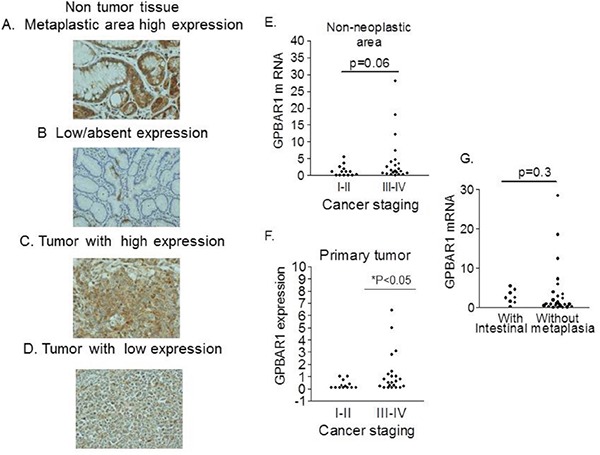
Immunohistochemistry **A-D.** The expression of GPBAR1 was examined in surgical samples obtained from patients who underwent surgery for the treatment of gastric cancer. GPBAR1 was expressed either in non-neoplastic and neoplastic tissues thought at different levels. **E.** No difference in GPBAR1 gene expression was detected in non-neoplastic tissue obtained from patients with stage I-II or stage III-IV disease (13 and 22 patients respectively) (E). In contrast, in primary tumor tissues **F.**, patients with stage III and IV (22 patients) had significantly higher expression of GPBAR1 mRNA in comparison with patients in stage I-II (13 patients) (p<0.05).) **G.** GPBAR1 mRNA was expressed at the similar level in areas with (8 patients) or without (26 patients) intestinal metaplasia.

We have first investigated the expression of the two major bile acid activated receptors, FXR and GPBAR1, in surgical samples obtained from patients who underwent surgery for the treatment of gastric cancer at the University Hospital of Perugia (Table 1). The expression of both receptors was then examined by immunohistochemistry and RT-PCR in pair tissue samples collected from macroscopically normal and neoplastic gastric mucosa. As illustrated in Figure 1A-1D, by immune-histochemistry we found that GPBAR1 was expressed either in non-neoplastic and neoplastic tissues and this finding was further confirmed by quantitative RT-PCR (Figure 1E–1G). As demonstrated in figure, the expression of GPBAR1 resulted slightly up-regulated, although not significantly, in the neoplastic tissue in comparison to healthy gastric mucosa. Further stratification of the patients demonstrated that the expression of the receptor was significantly higher in patients with advanced disease: indeed, patients with stage III and IV had significantly higher expression in comparison with patients in stage I-II (p<0.05). Taken together with the fact that such difference was not observed in non-neoplastic areas and that there was no difference among the two main histological phenotypes (i.e. intestinal vs diffuse), these data support the concept that development of more advanced disease associated with higher expression of GPBAR1. In contrast to GPBAR1, we found that expression of FXR, was reduced in neoplastic area compared with non-neoplastic tissues.

**Table 1:** Demographic data of 35 patients in primary gastric cancers

Sex	Males	22
	Female	13
Age	71, 25 y (range 50-89)	
		
Tumour Site	Residual Limb	1
	Body/Angulus	14
	Antrum	15
	Cardias	4
	Hep.-Gastr. Junction	1
		
Lymphnodes	D1	10
	D2	8
	D3	12
	D4	5
		
Histology	Diffuse	17
	Intestinal	10
	Intestinal+ diffuse	1
	Not specified	2
	Mucinous	3
	Signet-ring cells	1
	Adenosquamous	1
		
T (Size and spread of tumour)	T1a	2
	T1b	1
	T2	6
	T3	9
	T4	2
	T4a	13
	T4b	1
		
Stage	I	7
	II	7
	III	13
	IV	7
	Not specified	1

**The size and spread of the stomach tumour (T)**

TX means the main tumor (primary) cannot be assessed. T1 means the tumor has started to grow into the wall of the stomach. T1 is further divided into T1a and T1b. T1a means the tumor is within the inner layers of the stomach (the mucosa). And T1b means the tumor has grown through the mucosa and into a layer of supportive tissue called the submucosa. T2 means the tumour has grown into the muscle layer of the stomach. T3 means the tumour has grown into the outer lining of the stomach. T4 means the tumour has grown right through the outer lining of the stomach. It is further divided into T4a and T4b. T4a means the tumour has broken through the outer lining of the stomach wall. And T4b means it has grown through the stomach wall and into other organs or body structures nearby such as the liver, foodpipe (oesophagus) or abdominal wall.

### GPBAR1 is expressed by gastric cancer cell lines and drives a migratory phenotype

**Figure 2:**
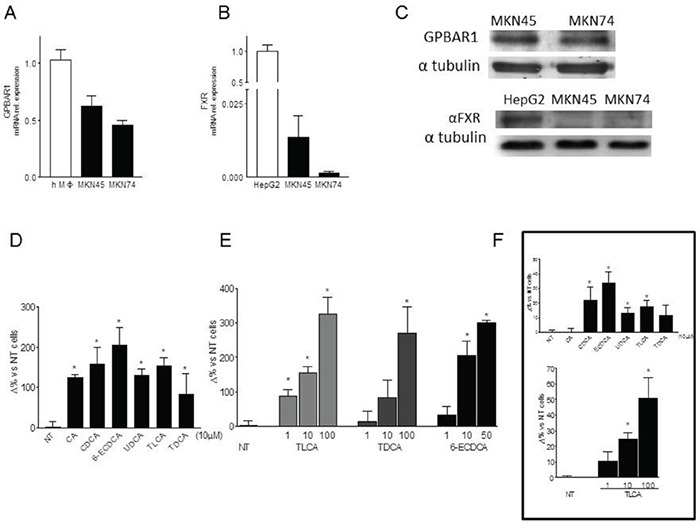
**A-B.** Expression levels of GPBAR1(A) and FXR(B) mRNA were evalueted by **ReaL-Time PCR** analysis in MKN45 and MKN74 cell lines. Values are normalized to GAPDH and are expressed relative to those of positive controls (PBMC isolated from an healthy donor and HepG2 cellular line respectively), which were arbitrarily settled to 1. The relative mRNA expression is expressed as 2(−ΔΔCt). **C.** Western Blot analysis of GPBAR1 and FXR protein expression in gastric cancer cell lines. Data was normalized with α-Tubulin expression. Western blot shown is representative of two others showing the same pattern. **D.** Transwell migration assay. MKN45 were seeded, serum starved and then primed with 10 μM of cholic acid (CA), chenodeoxycholic acid (CDCA), ursodeoxycholic acid (UDCA), taurolithocholic acid (TLCA), taurodeoxycholic acid (TDCA) and 6-ECDCA. *P<0.05 versus not treated (NT) **E.** In another experimental setting, cells were treated with TLCA (1, 10 and 100μM), TDCA (1, 10 and 100μM), 6-ECDCA (1, 10 and 50μM) for 72 hours. Experiments were performed in triplicate. All three ligands increased, in a dose-response manner, migration activity of gastric cells in comparison with control cells. *P<0.05 versus not treated (NT). **F.** Cell adhesion to peritoneum. Experiments were conducted in triplicate. TLCA treatment significantly increased cellular adhesiveness of gastric tumor cells to murine parietal peritoneum in a dose dependent manner. *P<0.05 versus not treated (NT).

Because these data suggest a role for GPBAR1 in determining a more aggressive phenotype, we have then investigated whether GPBAR1 was expressed in gastric cancer cell lines and its activation results in a migratory phenotype. For this purpose MKN45 and MKN74 human gastric cancer cell lines (respectively a poorly differentiated and a moderately differentiated gastric adenocarcinomas) were investigated. By RT-PCR analysis we found that both gastric cancer cell lines express high levels of GPBAR1 mRNA, ≈ 50% compared to PBMC used as positive control, (Figure 2A), with MKN45 cells showing a slightly higher expression of the receptor. These data were confirmed by Western Blot analysis (Figure 2C). In contrast to GPBAR1, gastric cancer cells did not express FXR protein, with MKN45 cells showing ≈ 100 folds lower expression of FXR mRNA in comparison to HepG2 cells (a human hepato-carcinoma cell line used as control).

To investigate whether exposure of gastric cancer cell lines to GPBAR1 ligands drives a migratory phenotype, transwell assays were performed using MKN45 cells. Cells were exposed to natural and synthetic bile acids: CA, CDCA, TLCA, TDCA and 6-ECDCA, a semy-sinthetic derivative of CDCA, (also known as INT-747 or obeticholic acid). All these agents have been shown to activate GPBAR1 [13]. As illustrated in Figure 2D, exposure to MKN45 cells to these agents at the concentration of 10 μM for 72 hours, resulted in a robust increase of migration as measured by invasion into Matrigel (P<0.05; Figure 2D 2C). The ability of bile acids to drive MKN45 migration into the transwell assay, was concentration dependent (Figure 2E).

Moreover, TLCA treatment of MKN45 cells significantly increased cellular adhesiveness to the murine parietal peritoneum in a dose dependent manner (Figure 2F).

**Figure 3:**
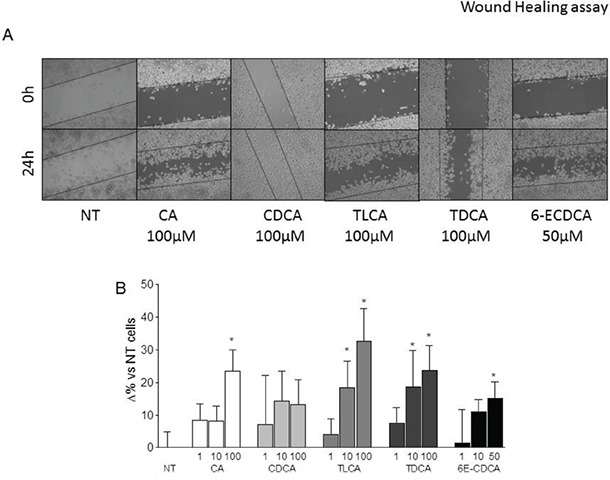
Wound healing assay **A-B.** Gastric cancer cells were serum starved and then primed with TLCA (1, 10 and 100 μM), TDCA (1, 10 and 100 μM), 6-ECDCA (1, 10 and 50 μM) for 72 hours. **(A)** A scratch wound healing assay is shown: MKN45 cell line left untreated or stimulated with 100 μM CA, CDCA, TLCA, TDCA and 50 μM 6E-CDCA. The images show cell migration after 72 hours of incubation with the indicated compound. After treatments with indicated compounds, cell monolayers were scraped in a straight line using a p200 pipette tip in order to create a “scratch”. A wound was generated and imaged at 0 an 24 hours with a phase-contrast. Images obtained from each samples at both time points were analyzed using Image J software and migration areas were expressed in pixels. All experiments were performed at least in triplicate. *P<0.05 versus not treated (NT).

Scratch wound healing is used to investigate the migration ability of cells *in vitro*. A wound was generated in MKN45 cell monolayers by a single scratch and the number of cells migrating in the scratch zone was compared at 0 and 48 h. Closure of the wound was significantly enhanced by pre-exposure of MKN45 cells to GPBAR1 ligands with TLCA and TDCA being more effective than CA and CDCA (Figure 3A and 3B). Again, as shown in Figure 3B, these effects were concentration dependent.

In contrast, activation of GPBAR1 by TDCA failed to induce resistance to two chemotherapeutic agents cisplatin and docetaxel (Supplementary Figure S1).

### Genomic effects of GPBAR1 activation in gastric cancer cells

**Figure 4:**
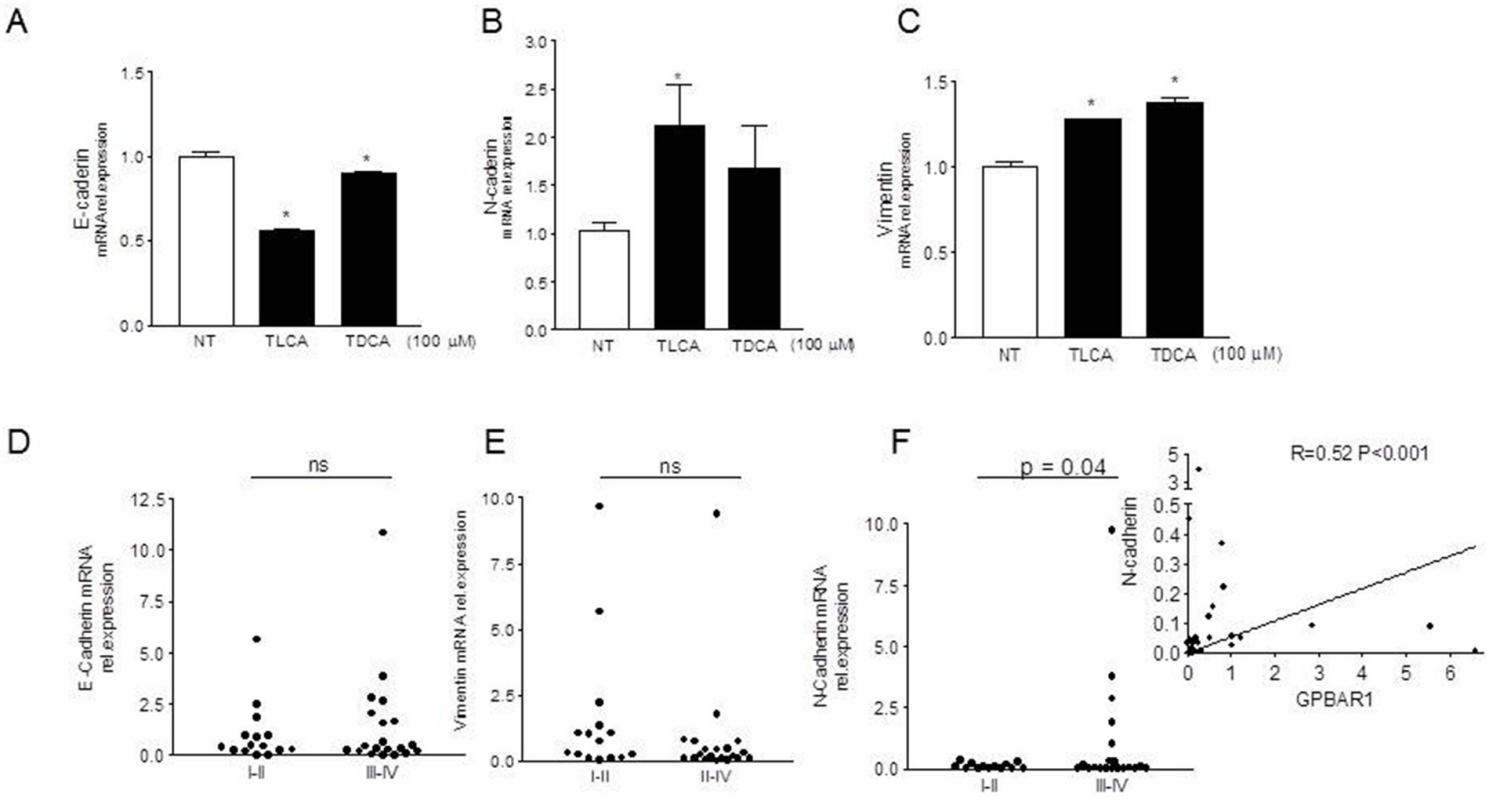
GPBAR1 agonists induce a EMT phenotype in MKN45 cells **A-C.** MKN45 cells were plated and after 24 hours of starvation were stimulated for 72 hours with TLCA and TDCA (100 μM). Exposing MKN45 cells to TLCA resulted in 50% percent reduction of E-cadherin mRNA (n= 4; P<0.05) **(A)**, ≈ 100% increase of N-cadherin (n=4; P<0.05) **(B)** and ≈ 30% increase of vimentin **(C)**. RT-PCR analysis of expression of E- **(D)** and N-cadherin **(F)** and vimentin **(E)** genes in gastric cancers (see figure 1). N-cadherin mRNA expression was found to be significantly higher in patients with advanced gastric cancers (stage III and IV)(p<0.05 versus stage I and II). (F inset) Correlation between N-Cadherin and GPBAR1 mRNA in specimens of human gastric cancers (n= 35 patients; r=0.52, p<0.001). Values are normalized to GAPDH and are expressed relative to those of positive controls, which are arbitrarily settled to 1. The relative mRNA expression is expressed as 2^(−ΔΔCt)^.

Having shown a potent pro-metastatic activity of GPBAR1 ligands in gastric cancer cells, as indicated by increased cells adhesion and migration activities, we have then investigated whether GPBAR1 activation induces phenotypic changes consistent with acquisition of a mesenchymal phenotype. Reduction of E cadherin and increased expression of N-cadherin along with vimentin are considered markers of EMT. As illustrated in Figure 4, we found that exposing MKN45 cells to TLCA at the concentration of 100 μM for 72 hours, results in 50% percent reduction of E-cadherin mRNA (n= 4; P<0.05) and robust increase of N-cadherin (n=4; P<0.05) and vimentin mRNA (n=4; P<0.05). Importantly a similar pattern of gene expression was found in the primary tumor of gastric cancer patients. Indeed, as shown in Figure 4F, while a robust increase in the expression of N-cadherin was detected in patients with advanced gastric cancer (p<0.05), no significant changes were found in terms of expression of E-cadherin or vimentin (Figure 4D and 4E).

**Figure 5:**
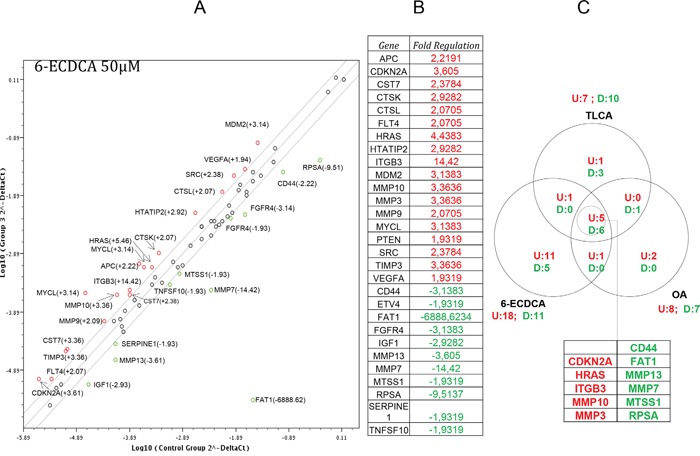
Gene array analysis of the expression of genes related to tumor metastasis in MKN45 cells exposed to selective and non-selective GPBAr1 ligands The cDNA used for the array was obtained from MKN45 cells (1×10^6^) plated, serum starved for 24 hours and then treated for 72 hours with 6-ECDCA 50μM. 6-ECDCA modulated the expression of 29 genes (18 upregulated and 11 downregulated). A comparative analysis of effects exerted by the three GPBAR1 agonists (Supplementary Figure S2 and S3), demonstrated that the three agents shared a regulatory effect on a small number of genes. Array analysis was carried out with the online software RT2 Profiler PCR Array Data Analysis (http://pcrdataanalysis.sabiosciences.com/pcr/arrayanalysis.php), Up-regulated/down-regulated genes were those genes whose expressions had been altered by more than 1.9 fold.

Because these data suggest that expression and activation of GPBAR1 contributes to a pro-metastatic phenotype effort were made to further characterize the effects of GPBAR1 ligation in MKN45 cells. For this purpose a gene array designed to analyze the expression of genes related to tumor metastasis was used. MKN45 were treated TLCA and oleanolic acid (a triterpenoid selective GPBAR1 agonist) and 6-ECDCA (a semisynthetic dual GPBAR1 and FXR ligand) for 72 hours. While none of the agents used reduced cell viability (data not shown), exposure to TLCA and oleanolic acid resulted in very comparable pattern of gene expression (Supplementary Figure S2 and S3). In contrast, 6-ECDCA modulated the expression of 29 genes (18 upregulated and 11 downregulated) (Figure 5). A comparative analysis of effects exerted by the three GPBAR1 agonists demonstrated that the three agents shared a regulatory effect on a small number of genes (Figure 5): 5 genes (CDKN2, HRAS, ITGB3, MMP3 and MMP10) were upregulated by more than 1.9-fold whereas 6 genes (CD44, FAT1, MMP7, MMP13, MTSS1 and RPSA) were down regulated in all three experimental groups by more than 1.9 folds, further confirming the potential for GPBAR1 to modulate the expression of genes involved in the acquisition of a pro-metastatic phenotype.

### Non genomic effects of GPBAR1 activation in gastric cancer cells

**Figure 6:**
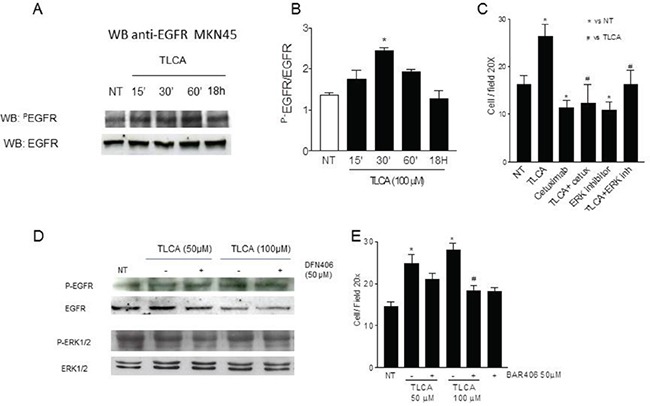
GPBAR1 activation by TLCA in MKN45 cells causes EGFR phosphorylation **A.** Cells treated with 100 μM TLCA were harvested at the indicated time. Cell lysates were immunoblotted with antibodies against phosphorylated EGFR (pEGFR) and total EGFR as indicated. **B.** Quantitative densitometry of the protein expression of the phosphorylated EGFR and total form. *<P0.05 versus not treated. **C.** Blockade of EGFR signaling abrogate the increased MKN45 migration activity TLCA-induced. MKN45 cells were treated with TLCA, the EFGR inhibitor cetuximab (alone or in combination with TLCA) and the MAPK inhibitor U0126. Data obtained are reported as Cell/Field 20x. **D.** MKN45 cells were incubated with 100 μM TLCA for 30′ alone or in combination with 50 μM DFN406, a synthetic GPBAR1 antagonist, and Western blotted with anti-EGFR, total and phosphorylated, and anti-Erk1/2, total and phosphorylated, antibodies. DFN406 prevented EGFR phosphorylation caused by TLCA and also prevented Erk1/2 phosphorylation. **E.** Blockade of GPBAR1 signaling by DFN406 abrogated the MKN45 migration caused by TLCA. Data obtained are reported as Cell/Field 20x. *P<0.05 versus not treated; #<0.05 versus TLCA.

Previous studies have shown that bile acids ability to promote the development of gastrointestinal malignancies [19] is mediated by the activation of the TACE/ADAM17 axis, resulting in EGFR cascade transactivation [30]. Thus, we have investigated whether gastric cancer cells stimulation with an agonist of GPBAR1 results in the phosphorylation of the EGFR. As shown in Figure 6A, exposure of MKN45 cells to TLCA resulted in EGFR phosphorylation that became apparent at 15 minutes of incubation and persisted up to 60 min. To investigate whether GPBAR1 mediated the effect of TLCA on EGF-R, MKN45 cells were incubated with DFN406, a synthetic bile acid endowed with GPBAR1 antagonistic activity (compound #3 in reference 28). As illustrated in Figure 6D, DFN406 prevented EGFR phosphorylation caused by TLCA and also prevented ERK1 and 2 phosphorylation. Additionally we found that DFN406 reduced MKN45 TLCA-induced migration in the transwell assay (Figure 6E). Because DFN406 has a specific action on the receptor, we can conclude that GPBAR1 is mechanistically involved in EGF-R phosphorylation caused by TLCA in gastric cancer cells.

To further investigate whether acquisition of a pro-metastatic phenotype, was mediated by EGF-R phosphorylation gastric cancer cells exposed to TLCA, were treated with cetuximab, an EGF-R inhibitor, or with U0126, an inhibitor of the ERK1 and ERK2. As illustrated in Figure 6C, exposure to cetuximab or U0126, *per se*, significantly reduced MKN45 migratory ability in comparison to control cells; moreover, both compounds completely abrogated the increased gastric cancer cells migration activity caused by TLCA (Figure 6C, n=4-5; p<0.05).

### GPBAR1 activation in gastric cancer cell lines associates with peritoneal spreading

**Figure 7:**
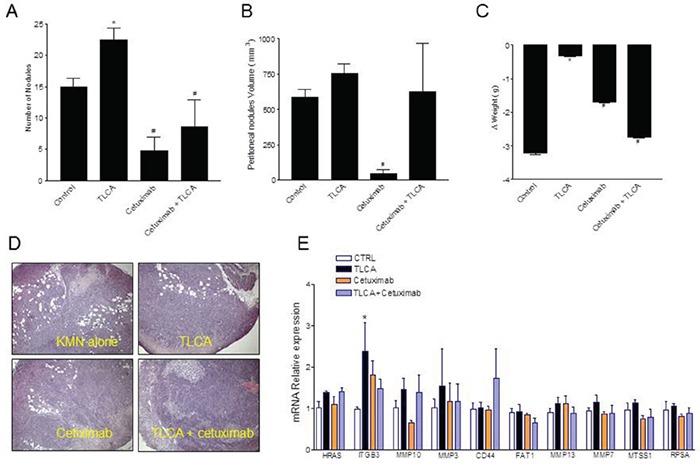
GPBAR1 activation enhances the metastatic potential of MKN45 cells **A-C.** MKN45 cells were pre-incubated with 100 μM TLCA and the injected i.p. in NOD-SCID mice (as described in materials and methods). TLCA pretreatment enhanced significantly the number and the volume of peritoneal nodules in comparison to control cells, Cetuximab reverted this pattern. *P<0.05 versus not treated; # P<0.05 versus TLCA. **(A-C)**. Ematoxylin and eosin staining of samples obtained from peritoneal nodules showing extensive necrosis in nodules exposed to cetuximab **(D)**. **E.** RT-PCR analysis of RNA extracted from peritoneal nodules showing a potent upregulation of ITGB3 in MKN45 cells pre-exposed to TLCA. Values are normalized to GAPDH and are expressed relative to those of positive controls, which are arbitrarily settled to 1. The relative mRNA expression is expressed as 2^(−ΔΔCt)^. *P<0.05 versus control.

Because the above mentioned data demonstrate that GPBAR1 activation causes a EMT and acquisition of a pro-metastatic phenotype, we have the investigated the role in cancer progression of the GPBAR1/EGFR signaling in a murine model of peritoneal carcinomatosis. MKN45 cells were left untreated or pretreated with TLCA, cetuximab or with the combination of both for 3 days and then implanted in the peritoneum of NOD-SCID mice. As shown in Figure 7A-7C, priming of MKN45 cells with TLCA significantly increased the number and the volume of peritoneal nodules in comparison to control cells, whereas the inhibition of EGF-R pathway by cetuximab almost completely abrogated MKN45 potential to form peritoneal nodules. Importantly, cetuximab effectively reverted the propensity of MKN45 cells toward generating peritoneal metastasis at least as assessed my measuring the number of peritoneal nodules (Figure 7A). However, peritoneal nodules in mice administered with cetuximab in combination with TLCA showed a large variability in nodule size. Accordingly, the H&E and eosin staining of samples obtained from peritoneal nodules demonstrates extensive necrosis in nodules exposed to cetuximab (Figure 7D–7E). Finally, RT-PCR analysis of mRNA obtained from peritoneal nodules lead to the demonstration that treatment of MKN45 with TLCA resulted in potent upregulation of ITGB3 and that this effect was markedly reduced by cetuximab (Figure 7E, n=5, P<0.05)

## DISCUSSION

Bile acids the end-product of cholesterol metabolism have been shown to signal through activation of variety of nuclear and cell surface receptors. By activating FXR, PXR and CAR, CDCA and CA elicits a series of genomic effects that have been deemed essential for regulation of lipid, cholesterol and bile acid homeostasis in intestine and liver. In addition, secondary bile acids, LCA and DCA, and their tauro- and glyco-conjugates, activate cell surface receptors, including TGR5 or M-BAR, a member of the rhodopsin-like superfamily of G protein coupled receptor (GPCR), recently christened as a GPBAR1 [10–14]. Similarly to other GPCRs, GPBAR1 senses molecules outside the cell and activate inside signal transduction pathways through agonist binding to an orthosteric binding site. GPBAR1 exerts both non-genomic effects (by increasing the intracellular concentration of cAMP) and genomic effects through the activation of cAMP-response element-binding protein, CREB, which binds to specific CREB response elements (CRE) in the promoter of target genes [10–13]. In contrast to FXR, GPBAR1 is highly expressed in the stomach and its activation attenuates gastric injury caused by aspirin and non-steroidal anti-inflammatory drugs. However, since duodenal reflux through the pylorus causes gastric adenocarcinoma in rodents and represents a risk factor for development of gastric adenocarcinoma in humans, a the receptor has attract a wide attention for its putative role in development of gastric adenocarcinoma [15–18].

In the present study we provide evidence that GPBAR1 is expressed in the normal gastric mucosa and that its expression, as measured by RT-PCR, increases significantly in tumor patients with advanced gastric adenocarcinomas (stage III and IV). By immuno-histochemistry GPBAR1 expression in non-neoplastic samples was found to be overexpressed in some patients with intestinal metaplasia [20] when compared with normal gastric epithelium (Figure 1A). However, the expression of the receptor was not constantly upregulated in gastric adenocarcinoma cells (intestinal and diffuse type), indicating that neither the presence of metaplasia or histologic subtype of the tumor was associated with a specific pattern of GPBAR1 gene expression.

Consistent with these findings, the quantitative PCR analysis demonstrates that: a) the levels of expression of GPBAR1 gene in cancer cells was similar among patients with or without intestinal metaplasia (Figure 1G), and b) that GPBAR1 mRNA levels did not differentiated among intestinal-type and diffuse-type cases. These findings seems to rule out a role for the intestinal metaplasia in regulating the expression of the receptor. In contrast, the expression of the receptor was significantly higher in patients with stage III-IV adenocarcinomas, in comparison to patients with stage I and II in both non-neoplastic and neoplastic samples (Figure 1E and 1F). Thus, GPBAR1 could be a marker of tumor aggressiveness. Consistent with this view, Cao et al. [20] have shown that moderate to strong GPBAR1 staining in gastric adenocarcinomas associates with decreased patient survival.

Because GPCRs are known to regulate cell migration, proliferation, differentiation and survival and play a mechanistic role in the development and progression of several cancers [30], we have then investigated whether GPBAR1 exerts regulate effects on gastric cancer cell lines. Previous studies have reported that GPBAR1 (TGR5) in AGS cell line [19], and we now show that two additional gastric cancer cell lines, MKN45 and MKN74, express the gene. Importantly, as illustrated in Figure 2, the other bile acid activated receptor, FXR, was essentially undetectable in these cell lines.

Cancer metastasis is a multi-step cascade that starts with EMT, dissociation from the primary tumor, and distal invasion [25]. Therefore, we have assessed the invasive capacity of MKN45 cells expressing GPBAR1 in response to natural and synthetic bile acids. Potent GPBAR1 ligands, 6-ECDCA and TLCA and TDCA effectively induced the acquisition of a migratory phenotype, including greater invasion into Matrigel and migration into scratch wounds and across transwell barriers and adhesion to mouse peritoneum. In contrast, weak GPBAR1 ligand, such as UDCA, were significantly less effective in inducing cell migration in all the three systems. The fact that bile acids effectively drive the acquisition of a pro-metastatic phenotype with the same rank of potency required for activation of GPBAR1 and that migration is inhibited by DFN406 a GPBAR1 antagonist [28] is a further evidence that support the role of this receptor [12, 13].

To produce metastasis carcinoma cells must temporarily lose defining features, such as cell–cell adhesion, epithelial tight junction, and desmosomes. Recent studies have shown that the typical EMT profile is correlated with tumor grade and metastasis of gastric cancer [25]. Thus, loss of expression of epithelial proteins such as E-cadherin and acquisition of mesenchymal proteins, such as N-cadherin and vimentin, associates with advanced stage and poor outcome in gastric carcinomas [25]. The results shown in Figure 4, demonstrate that exposure of MKN45 cells to bile acids causes the acquisition of a mesenchymal phenotype as indicated by reduced E-cadherin expression and increased N-cadherin and vimentin. Importantly, analysis of expression of these three genes in tumor samples from 35 patients confirms these findings, since the expression of N-cadherin was significantly increased in patients with stage III-IV in comparison with patients with stage I-II, and correlates, in a highly significant manner, with the expression of GPBAR1 in the tumors (Figure 4D), further confirming the fact that expression of GPBAR1 correlates with advanced disease and poor prognosis [20, 25].

Because targeting EMT is a promising approach to protect from metastasis formation [25], we have further investigated whether activation of GPBAR1 induces changes in the expression of a subset of genes known to be involved in tumor diffusion. The data presented in Figure 5 demonstrate that activation of GPBAR1 with three different ligands results in a profound alteration of gene expression in MKN45 cells. Indeed, by using an array that investigate 80 genes, we found that 6-ECDCA a potent dual FXR/GPBAR1 ligand causes the upregulation or downregulation of 29 gene. In contrast, TLCA and oleanolic acid [13] alter the expression of 17 and 15 genes. Analysis of genes that were up- or down-regulated by the three treatments lead to the identification of 11 genes that were regulated at least by two-folds by all treatments. Thus, activation of GPBAR1 leads to the upregulation of 5 genes (CDKN2A, HRAS, IFGB3, MMP10 and MMP3) and down regulation of 6 genes (CD44, FAT1, MMP13, MMP7, MTSS1 and RPSA). By functional analysis all these genes were clustered in pathways related to tumor metastasis. Specifically, we found that while GPBAR1 activation reduced the expression of MMP7 and MMP13 genes, the expression of two other metalloproteinases MMP3 and MMP10 was robustly enhanced by all there agonists [31–33]. Further on, all three GPBAR1 ligands were effective in inducing the expression of ITGB3 [34], a integrin beta-chain subunit coded by a serotonin-related gene on chromosome 17, that has been reported to be associated with the risk of several human cancers, including colorectal cancer. The ITGB3 family includes αIIb β3 and αvβ3. αIIb β3 mainly expresses on the surface of platelets and megakaryocytes, while αvβ3 mainly expresses on the surface of endothelial cells, smooth muscle cells, monocytes, and platelets [35]. Because previous studies coupled ITGB3 to EMT and metastasis, and silencing of ITGB3 by a variety of approach has been demonstrated to rescue from metastasis formation in several cancer models, our study suggest that targeting this integrin could be useful in the treatment of gastric cancer.

In addition to these genomic effects, GPBAR1 activation causes non-genomic effects that are partially mediated by phosphorylation of EGF-R [31]. Indeed, exposure of MKN45 cells to GPBAR1 ligands results in a time dependent EFG-R phosphorylation. The effect was prevented by treating the cells with DFN406, a GPBAR1 antagonist [28, 31]. Exposure of MKN45 cells to DFN406, not only inhibited the phosphorylation of the EGF-R but also reduces signaling down-stream to the receptor by attenuating ERK1 and 2 phosphorylation and transwell migration of cells exposed to TLCA.

In addition, inhibition of EGF-R phosphorylation by cetuximab [36, 37], inhibited transmigration of MKN45 cells pre-treated with TLCA *in vitro* but was also effective in protecting against peritoneal spreading caused by TLCA pre-treatment in a xenograph model of peritoneal carcinogenesis. In this model, implanting MKN45 cells that were pre-exposed to TLCA resulted in development of a diffuse disease that was markedly attenuated by treating the cells with cetuximab, further confirming the role EFG-R in mediating the pro-metastatic activity of TLCA. Analysis of genes in peritoneal nodules confirmed that TLCA treatment results in a robust induction of ITGB3, a pattern that was reversed by treating the cells with cetuximab. Taken together these data suggest that regulation of ITGB3 by TLCA could be due to both genomic and non-genomic effects.

In conclusion, we have provided evidence that advanced gastric cancer are characterized by high expression of the bile acid receptor GPBAR1 and that expression of this receptor strongly correlated with that of N-cadherin. In *in vitro* experiments we have shown that activation of GPBAR1 in gastric cancer cells trigger the EMT and acquisition of aggressive phenotype. These effects are mediated by regulation of several genes, including ITGB3, by both genomic and non-genomic effects. Present results highlight the potential of GPBAR1 antagonist in the management of advanced gastric cancer.

## MATERIALS AND METHODS

### Patients and specimens

Gastric carcinoma tissues were obtained from 35 gastric cancer patients (22 males and 13 females) treated by surgical resection at the Department of Surgery, Santa Maria della Misericordia Hospital (Italy). Surgeries were conducted from August 2014 to December 2015. The patients mean age was 71.25 years (range: 50 to 89 years). None of the patients received chemotherapy or radiation before surgery. Permission to collect post-surgical samples was granted to Prof. Fiorucci by the ethical committee of Umbria (CEAS). Permit FI00001, n. 2266/2014 granted on February 19, 2014. An informed written consent was obtained by each patient before surgery. Accurate clinical information and pathologic diagnosis were available for all patients. Disease staging was defined according to the TNM staging system of the American Joint Committee on Cancer [26]. The tumors (Table 1) were divided according to guidelines in Stage I (7 cases), II (7 cases), III (13 cases) and IV (8 cases) and into diffuse and intestinal sub-types according to the Lauren Classification [27].

### Cell lines

HepG2 cells were purchased from American Type Culture Collection (ATCC, Promochem, Milan, Italy). MKN74 and MKN45 were from the Japanese Collection of Research Bioresources (Human Science Research Resources Bank, Osaka, Japan). The two gastric cell lines were maintained in RPMI cell culture medium supplemented with 10% FBS, 1% penicillin/streptomycin in a humidified atmosphere of 5% CO_2_ in air, at 37°C. HepG2 cells were maintained in E-MEM (Eagle's minimal essential medium) cell culture medium supplemented with 10% FBS, 1% penicillin/streptomycin in a humidified atmosphere of 5% CO_2_ in air, at 37°C. Cells were regularly passaged to maintain exponential growth. Peripheral whole blood sample (~ 30 ml) from an healthy donor was withdrawn in vacutainer tubes containing EDTA. PBMC were first isolated by density gradient centrifugation using the Hystopaque reagent (Pharmacia Biotech) and then positively selected using CD14 magnetic beads and LS columns according to the manufacturer's instructions (Miltenyi Biotec). After isolation monocytes were lysed with 1 ml TRIzol reagent (Invitrogen).

### Cell migration assay

MKN45 cells (5×10^5^/well) were seeded in a 6-well plate; on day 2, cells were serum starved and then primed with TLCA(1, 10 and 100μM), TDCA (1, 10 and 100μM), 6-ECDCA (1, 10 and 50μM) for 72 hours. In an another experimental setting, cells were treated with 10μM of CA, CDCA, UDCA, TLCA, TDCA and 6-ECDCA. In order to investigate GPBAR1 ability to activate EGF Receptor signaling, MKN45 cells were treated with cetuximab 200 μg/ml (alone or in combination with TLCA 100μM) and the MEK 1/2 inhibitor U0126 50μM (alone and in combination with TLCA 100μM) for 72 hours. Finally in order to investigate whether GPBAR1 mediated the effect of TLCA on EGFR, MKN45 cells were treated with TLCA (5-100 μM), alone or in combination with DFN406 (50 μM), a GPBAR1 antagonist. All treatments were performed in Serum Free Medium condition. The Transwell® Permeable Supports (Corning, USA) were used for this assay as recommended by the manufacturer. After the incubation period, gastric cancer cells were detached and re-suspended at 2.5×10^5^ cells/ml; 200 μl of cell suspensions were added in the upper chamber in absence of serum; the lower chamber was filled with culture medium containing 10% FBS as a chemoattractant. After 24 h of incubation, non-invading cells on the upper surface of the membrane were removed by scraping. Invading cells on the lower side of the membrane were fixed methanol and stained with 0.1% Toluidine blue solution and counted under a light microscope (200×/10 fields for well). Experiments were performed in triplicate.

### Cell adhesion to peritoneum

MKN45 cells were plated onto complete RPMI medium; on day 2, cells were starved and left untreated or triggered with TLCA (1, 10 and 100μM) for 72 hours. In an another experimental setting, cells were treated with 10μM of CA, CDCA, TLCA, TDCA and 6-ECDCA for 72 hours. On day 5, excised parietal peritoneum (~1.6 cm^2^) was placed in a 24-well culture plate, which had been filled with 1.0 ml of 1% BSA/RPMI 1640. Gastric cancer cells were detached, fluorescently labeled with BCECF-AM (5 μM) at 37°C for 30 minutes and washed twice with 1% BSA/RPMI 1640. After trypan blue staining, 500μl of a suspension of living cells (5 × 10^5^ cells/ml in 1% BSA/RPMI 1640) were overlaid on the peritoneum in a 24-well plate, and the plate was incubated at 37°C for 60 minutes [28]. After gentle washing with PBS, the cells adherent to the peritoneum were lysed with 1.0 ml of TRIS 50 mM plus SDS 1%; fluorescence intensity was measured with a fluorescence spectrophotometer (Ex = 490 nm and Em = 520 nm). Experiments were conducted in triplicate.

### Wound healing assay

MKN45 cells were seeded in RPMI complete medium in 12 well-plate at 5×10^5^ cells/well; on day 2 cells were primed for 72 hours with increasing concentrations of CA, CDCA, TLCA, TDCA and 6- (1, 10 and 50μM) for 72 hours. In an another experimental setting, cells were treated with 10μM of CA, CDCA, TLCA, TDCA and 6-ECDCA. After treatments, the cell monolayers were scraped in a straight line using a p200 pipette tip in order to create a “scratch”; cell debris were removed by washing with PBS and then fresh medium was added to each well. Immediately after scratch creation, plate was placed under a phase-contrast microscope and a first image of the scratch was acquired [29]. Plate was then placed in tissue culture incubator at 37°C for 24 hours, after that a second image of each scratch was acquired. Images obtained from each samples at both time points were analyzed using Image J software and migration areas were expressed in pixels. All experiments were performed at least in triplicate.

### *In vivo* tumorigenesis

Eight-week-old male NOD-SCID mice (supplied by the animal center of the University of Perugia) were housed under pathogen-free conditions. The care and use of the animals were approved by the Institutional Animal Care and Use Committee of the University of Perugia and were in accordance to European guidelines for care of experimental animals. Protocols were approved by the Istituto Superiore di Sanita (Italy) and approval granted to Prof. Annibale Donini (No. 208/2009/b and 41/2014b). To generate models of experimental peritoneal carcinomatosis, MKN45 cells were injected intra-peritoneal in NOD-SCID mice, as described previously [30]. To examine the potential role of the GPABR1/EGFR signaling in peritoneal carcinomatosis, before inoculation MKN45 cells were treated for 72 hours with TLCA 100 μM, cetuximab 200 μg/ml or with the combination of both drugs. For each treatment, a cell suspension of 1×10^7^ cells in a total volume of 0.2 ml of medium without serum was injected into the peritoneal cavity (i.p.) of each mouse using a 23-gauge (N=5 per group). The extent of peritoneal carcinogenesis was evaluated on day 10 by necroscopy: numbers and volume of peritoneal nodules were evaluated.

### Western blotting

MKN45 and MKN74 cells were lysed and proteins (50 μg) were separated by 10% SDS- polyacrylamide gels, followed by electro-transfer onto a nitrocellulose membrane. For EGFR and Erk1/2 western blotting only MKN45 cells were lysed and proteins (50 μg) were separated by 8% and 12% SDS-polyacrylamide gels respectively, followed by electro-transfer onto a nitrocellulose membrane. The membranes were sequentially incubated with blocking buffer (TBS-Tween containing 5% nonfat dry milk or 5% BSA respectively) for 1 hour at room temperature, and then overnight at 4°C with one of the following antibodies: anti-TGR5 (1:2000, #ab72608 Abcam), anti-FXR (1:2000 #sc-13063 Santa Cruz Biotechnology), anti-EGFR (1:1000, #2232 Cell Signaling), and anti-phospho-EGFR antibody (1:1000, #4407 Cell Signaling), anti-Erk1/2 (P44/42 MAPK) (1:1000, #46955 Cell Signaling), anti-phospho-Erk1/2 (p-P44/42 MAPK) (1:1000, #91015 Cell Signaling). Primary antibody were detected with the horseradish peroxidase (HRP)-labeled secondary antibodies (1:10000, BioRad) for 1 hour at room temperature. After washing with TBST, protein bands were visualized by Lite Ablot TURBO (Euroclone) according to the manufacturer's instructions.

### Polymerase chain reaction array analysis

MKN45 cells (1×10^6^) were plated onto 10-cm culture dish; on day 2, cells were serum starved for 24 hours and then treated for 72 hours with TLCA 100μM, the semi-synthetic derivative of CDCA, the 6-ECDCA 50μM and Oleanolic Acid 25μM. Total RNA was prepared from each specimen using TRIzol reagent (Invitrogen) and revere transcribed with SuperScript II Reverse Transcriptase (Invitrogen) following the manufacturer's instructions. A total of 10 ng of cDNA was pipetted into each well of a 96-well gene array plate [Human Tumor Metastasis RT2 Profiler PCR Array, Qiagen (http://www.sabiosciences.com/rt_pcr_product/HTML/PAHS-028Z.html)] and amplified following the manufacturer's instructions. This gene array is designed to assess 84 genes, known to be involved in metastasis, that encode for proteins involved in cell adhesion, cell cycle progression, cell growth and proliferation, apoptosis, components of extracellular matrix, transcription factors and regulators, and other genes related to tumor metastasis.

### Real-time PCR

Total RNA was isolated from MKN45 or tissue using the TRIzol reagent according to the manufacturer's specifications (Life Technologies). One microgram of RNA was purified from genomic DNA by DNase-I treatment (Life Technologies) and reverse-transcribed using random hexamer primers with Superscript-II (Life Technologies) in a 20 μL reaction volume. Twenty-five ng cDNA were amplified in a 20 μL solution containing 200 nM of each primer and 10 μL of 2X SYBR FAST Universal ready mix (Invitrogen). All reactions were performed in triplicate, and the thermal cycling conditions were as follows: 10 min at 95°C, followed by 40 cycles of 95°C for 15 s, 56°C for 20 s and 72°C for 30 s in StepOnePlus instrument (Applied Biosystems). The relative mRNA expression was calculated accordingly to the Ct method. PCR primers were designed using the software PRIMER3 (http://frodo.wi.mit.edu/primer3/) using published data obtained from the NCBI database. Forward and reverse primer sequences were as follows: hGAPDH:gaaggtgaaggtcggagt and catgggtggaatcatattggaa;hTGR5:cactgttgtccctcctctcc and acactgctttggctgcttg; hFXR: tacatgcgaagaaagtgtcaaga and actgtcttcattcacggtctgat; hVimentin: gagaactttgccgttgaagcand tccagcagcttcctgtaggt; hE-cadherin: gaatgacaacaagcccgaat and tgaggatggtgtaagcgatg; hN-cadherin: aggtttgccagtgtgactcc and gatgatgatgcagagcagga; hHRAS: agctgatccagaaccattttgt and gttgatggcaaacacacacag; hITGB3: gggctgatgactgagaagctat and tcccataagcatcaacaatgag; hMMP3: tgatgttggtcacttcagaacc and ttcccagactttcagagctttc; hMMP10: tcatttgatttctgcattttgg and cctgcttgtacctcatttcctc; hCD44: gaagatttggacaggacaggac and ccttcagaatgatttgggtctc; hFAT1: aaagaagatgcacctgttggtt and tccagtcgatctgacgtctcta; hMMP13: cctggacaagtagttccaaagg and ccttgtacatcgtcatcaggaa; hMMP7: aataatgcagaagcccagatgt and cgatccactgtaatatgcggta; hMTSS1: ctaggggaaataacccaccttc and caagtccagaatcacctgttca; hRPSA: cttcactcctggaaccttcact and tacacagcgcaatggtaggtag.

### Propidium iodide staining

Gastric cancer cell lines, MKN45 and MKN74, plated at 1×10^6^ cells/well were pre-treated with 6- ECDCA (5 μM) for 3 hours and then challenged with cisplatin (5 and 30 μM), docetaxel (1.5 μM) and 5-fluorouracil (4 μM) for 24 hr. Cells were trypsinized, washed twice with PBS and fixed with 70% cold ethanol overnight at −20°C. Subsequently, cells were centrifuged, stained with propidium- iodide solution, washed with PBS and detection of apoptotic cells was assessed by flow citometry analysis. Experiment was performed in quadruplicate.

### Statistical analysis

The Pearson rank correlation analysis was applied to assess the association between the expressions of vimentin, E- and N-cadherin with GPBAR1. Data are expressed as means ± SE. For cells and animal studies, statistical difference between two groups were determined by Student's *t-*test. Differences between multiple groups were tested using ANOVA followed by Bonferroni. Two-tailed *P* values of 0.05 or less were considered to be statistically significant. For statistical analysis of data shown in Figure 5, the array analysis was carried out with the online software RT2 Profiler PCR Array Data Analysis (http://pcrdataanalysis.sabiosciences.com/pcr/arrayanalysis.php). Up-regulated/down-regulated genes were those genes whose expressions had been altered by more than 1.9 fold.

## SUPPLEMENTARY MATERIALS FIGURES AND TABLES


