# Supplementary material for: The bile acid receptor GPBAR1 (TGR5) is expressed in human gastric cancers and promotes epithelial-mesenchymal transition in gastric cancer cell lines
Source: Oncotarget. 2016 Jul 7;7(38):61021–35. doi: 10.18632/oncotarget.10477 (PMC5308633; doi:10.18632/oncotarget.10477)
Supplement: Supplementary file 1 [file oncotarget-07-61021-s001.pdf]

The bile acid receptor GPBAR1 (TGR5) is expressed in human gastric cancers and promotes epithelial-mesenchymal transition in gastric cancer cell lines

SUPPLEMENTARY INFORMATION

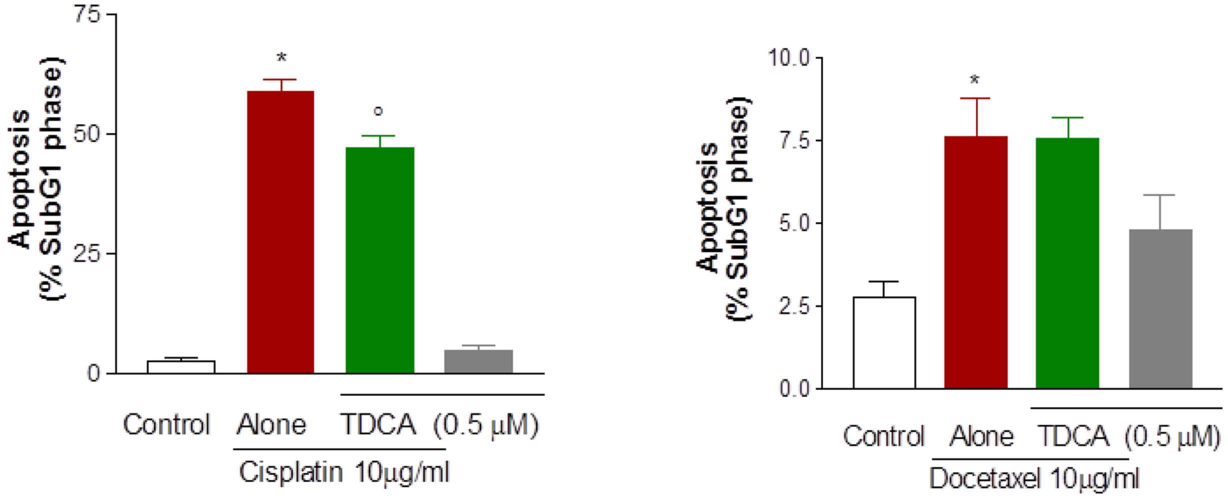

Supplementary Figure S1: MKN-45 cells were plated at 1x10⁶cells/well, and pre-treated with TDCA (0.5 µM) for 3 hours and then challenged with Cisplatin (10 µg/ml) or Docetaxel (10 µg/ml) for 24 hr. Cells were stained with propidium-iodide solution and percentage of apoptotic cells assessed by flow cytometry.

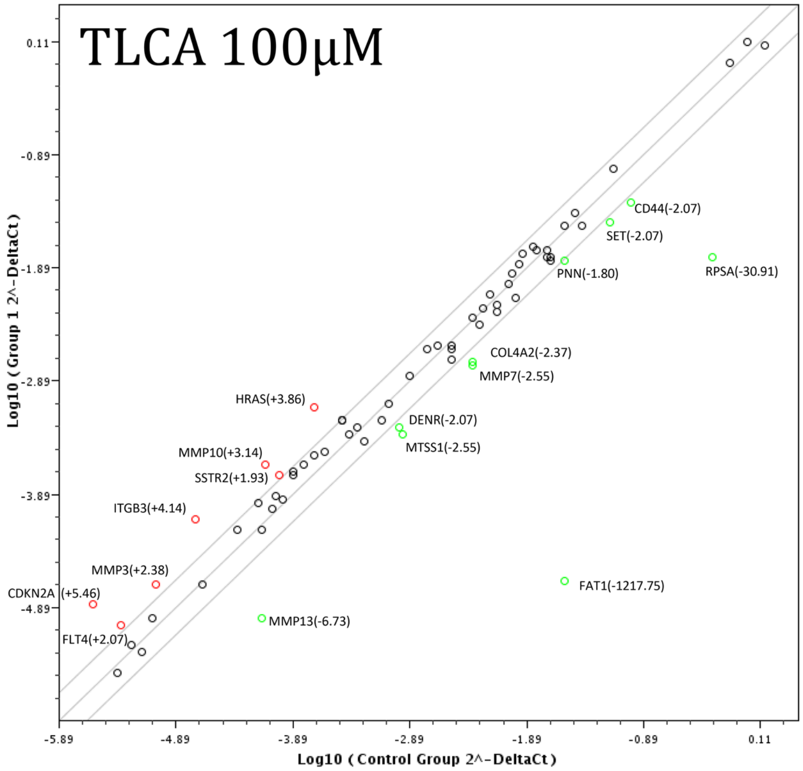

Gene	*Fold Regulation*
CDKN2A	5,4642
FLT4	2,0705
HRAS	3,8637
ITGB3	4,1411
MMP10	3,1383
MMP3	2,3784
SSTR2	1,9319
CD44	-2,0705
COL4A2	-2,3784
DENR	-2,0705
FAT1	-1217,7481
MMP13	-6,7272
MMP7	-2,5491
MTSS1	-2,5491
PNN	-1,8025
RPSA	-30,91
SET	-2,0705

2

Supplementary Figure S2: MKN45 cells (1x10^6) were plated, serum starved for 24 hours and then treated for 72 hours with TLCA 100μM. The cDNA obtained from this cells was used to analyze the expression of genes related to tumor metastasis by a gne array. Array analysis was carried out with the online software RT2 Profiler PCR Array Data Analysis (http://pcrdataanalysis.sabiosciences.com/pcr/arrayanalysis.php). Up-regulated/down-regulated genes were those genes whose expressions had been altered by more than 1,9 fold.

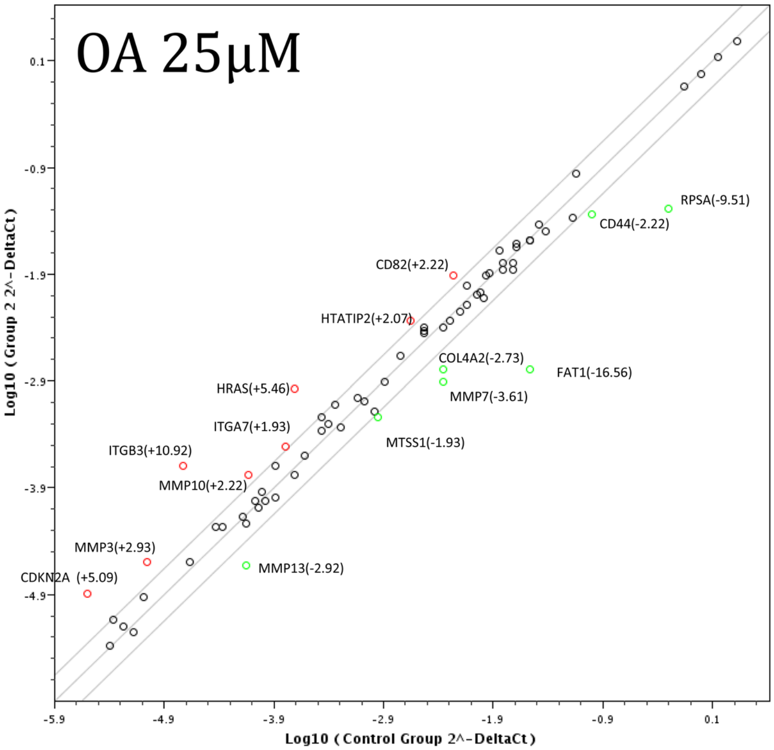

Gene	*Fold Regulation*
CD82	2,2191
CDKN2A	5,0982
HRAS	5,4642
HTATIP2	2,0705
ITGA7	1,9319
ITGB3	10,9283
MMP10	2,2191
MMP3	2,9282
CD44	-2,2191
COL4A2	-2,7321
FAT1	-16,5642
MMP13	-2,9282
MMP7	-3,605
MTSS1	-1,9319
RPSA	-9,5137

Supplementary Figure S3: MKN45 cells (1x10[6]) were plated, serum starved for 24 hours and then treated for 72 hours with OA 25µM. The cDNA obtained from this cells was used to analyze the expression of genes related to tumor metastasis by a gne array. Array analysis was carried out with the online software RT2 Profiler PCR Array Data Analysis (http://pcrdataanalysis.sabiosciences.com/pcr/arrayanalysis.php). Up-regulated/down-regulated genes were those genes whose expressions had been altered by more than 1,9 fold.
